# Supplementary material for: Identification of photoperiod-induced specific miRNAs in the adrenal glands of Sunite sheep (Ovis aries)
Source: Front Vet Sci. 2022 Jul 22;9:888207. doi: 10.3389/fvets.2022.888207 (PMC9354845; doi:10.3389/fvets.2022.888207)
Supplement: Supplementary file 1 [file Data_Sheet_1.ZIP › Supplementary file/miRDeep2 file.zip!/miRDeep2 file/Novel_Structure/SP42A2/Novel_663.pdf]

Provisional ID	:	NC_019481.2_40362
Score total	:	1661.5
Score for star read(s)	:	3.9
Score for read counts	:	1654
Score for mfe	:	2
Score for randfold	:	1.6
Score for cons. seed	:	
Total read count	:	3256
Mature read count	:	3245
Loop read count	:	0
Star read count	:	11

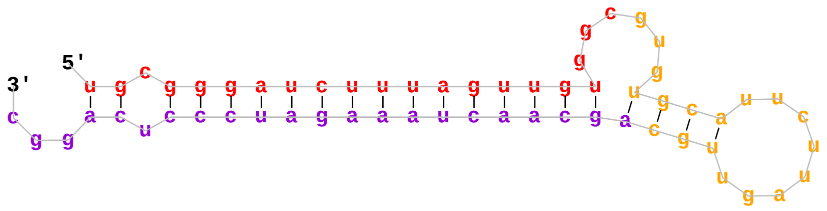

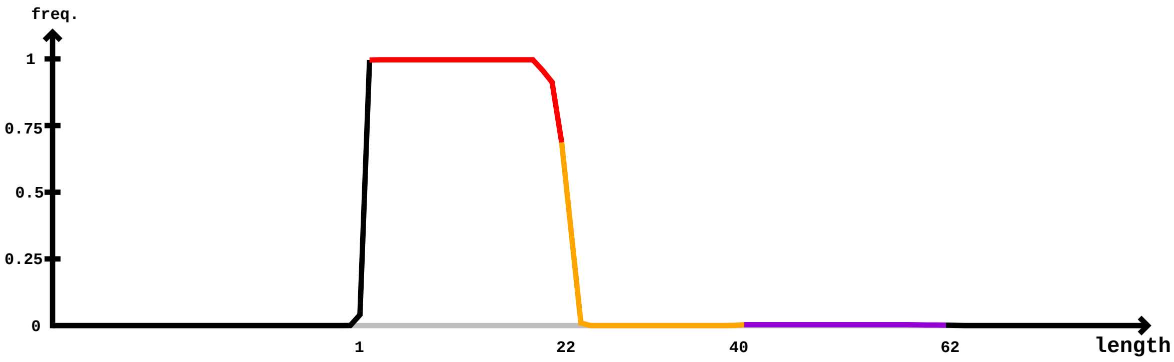

Mature Star

```
5'- auaacccauaaaagcagccgacguugcggcaugcgggaucuuuaguuguggcgugugcauucuuaguugcagcaacuaaagaucccucaggcugcaaugaagaucagagauc  -3'  obs
    auaacccauaaaagcagccgacguugcggcaugcgggaucuuuaguuguggcgugugcauucuuaguugcagcaacuaaagaucccucaggcugcaaugaagaucagagauc       exp
    .....................(((((((((.((.(((((((((((((((......((((........))))))))))))))))))).)).)))))))))..((((...))))   reads   mm   sample
    .............................caugcgggaucuuuaguuguA..............................................................   1       1    seq
    ..............................augcgggaucuuuaguugu...............................................................   10      0    seq
    ..............................augcgggaucuuuaguuguA..............................................................   3       1    seq
    ..............................augcgggaucuuuaguugug..............................................................   6       0    seq
    ..............................augcgggaucuuuaguugugU.............................................................   2       1    seq
    ..............................augcgggaucuuuaguugugA.............................................................   11      1    seq
    ..............................augcgggaucuuuaguuguggc............................................................   55      0    seq
    ..............................augcgggaucuuuaguuguggU............................................................   22      1    seq
    ..............................augGgggaucuuuaguuguggc............................................................   1       1    seq
    ..............................augcgggaucuuuaguuguggcg...........................................................   9       0    seq
    ..............................augcgggaucuuuaguuguggcA...........................................................   12      1    seq
    ...............................ugUgggaucuuuaguugu...............................................................   1       1    seq
    ...............................ugcAggaucuuuaguugu...............................................................   1       1    seq
    ...............................ugcgAgaucuuuaguugu...............................................................   1       1    seq
    ...............................ugcgggaucuuuaguugu...............................................................   100     0    seq
    ...............................ugAgggaucuuuaguugu...............................................................   1       1    seq
    ...............................ugcgggaucuuuaguugC...............................................................   12      1    seq
    ...............................ugcgggaucuuuaguugA...............................................................   1       1    seq
    ...............................ugcgggaucuuuaguuguA..............................................................   31      1    seq
    ...............................ugcgggaucuuuaguugug..............................................................   105     0    seq
    ...............................ugcgggaucuuuUguugugg.............................................................   1       1    seq
    ...............................ugcgggaucuuuaguugugg.............................................................   497     0    seq
    ...............................ugcgggaucuuuaguugCgg.............................................................   3       1    seq
    ...............................ugcgggaucuuuaguugugA.............................................................   180     1    seq
    ...............................ugcgggaucuuuaguuguAg.............................................................   2       1    seq
    ...............................ugcgggaucuuuaguugAgg.............................................................   1       1    seq
    ...............................ugcgAgaucuuuaguugugg.............................................................   5       1    seq
    ...............................ugcgggaucuuuaguugugU.............................................................   34      1    seq
    ...............................ugcgggaucuuuaguuguAgc............................................................   2       1    seq
    ...............................ugcgggaucuuuaguuguggc............................................................   1418    0    seq
    ...............................ugcgggaucuuuaguugugAc............................................................   2       1    seq
    ...............................ugcgggaucuuuaguugugUc............................................................   2       1    seq
    ...............................ugcgggaucuuuaguuguggU............................................................   552     1    seq
```

```
                               Mature                                Star
auaacccauaaaagcagccgacguugcggcaugcgggaucuuuaguuguggcgugugcauucuuaguugcagcaacuaaagaucccucaggcugcaaugaagaucagagauc
...............................ugcgggaucuuuaguugCggc............................................................   4    1    seq
...............................ugUgggaucuuuaguuguggc............................................................   5    1    seq
...............................ugcggAaucuuuaguuguggc............................................................   1    1    seq
...............................ugcgggaucuuuagCuguggcg...........................................................   12   1    seq
...............................ugcAggaucuuuaguuguggcg...........................................................   41   1    seq
...............................ugcgggGucuuuaguuguggcg...........................................................   11   1    seq
...............................ugUgggaucuuuaguuguggcg...........................................................   19   1    seq
...............................ugcUggaucuuuaguuguggcg...........................................................   4    1    seq
...............................ugcgggaucuuuaguuguggUg...........................................................   30   1    seq
...............................ugcUggaucuuuaguuguggcgu..........................................................   1    1    seq
...............................ugcgggaucuuuagCuguggcgu..........................................................   3    1    seq
...............................ugUgggaucuuuaguuguggcgu..........................................................   6    1    seq
...............................ugcgggGucuuuaguuguggcgu..........................................................   9    1    seq
...............................ugcgAgaucuuuaguuguggcgu..........................................................   12   1    seq
................................gcgggaucuuuaguugugg.............................................................   1    0    seq
................................gUgggaucuuuaguuguggc............................................................   1    1    seq
................................gcUggaucuuuaguuguggcg...........................................................   1    1    seq
.....................................................................cCgcaacuaaagaucccuc........................   2    1    seq
......................................................................agcaacuaaagaucccuca.......................   2    0    seq
......................................................................agcaacuaaagaucccucagg.....................   2    0    seq
......................................................................agcaacuaaagaucccucaggc....................   5    0    seq
```
